# Investigation on the Influence of Humidity on Stimulated Brillouin Backscattering in Perfluorinated Polymer Optical Fibers

**DOI:** 10.3390/s18113952

**Published:** 2018-11-15

**Authors:** Andy Schreier, Sascha Liehr, Aleksander Wosniok, Katerina Krebber

**Affiliations:** Devision Fibre Optic Sensors, Bundesanstalt für Materialforschung und -prüfung (BAM), Unter den Eichen 87, 12205 Berlin, Germany; sascha.liehr@bam.de (S.L.); aleksander.wosniok@bam.de (A.W.); katerina.krebber@bam.de (K.K.)

**Keywords:** polymer optical fiber, CYTOP, backscattering, stimulated Brillouin scattering, Brillouin frequency shift, Rayleigh backscattering, humidity, relative humidity

## Abstract

In this paper perfluorinated graded-index polymer optical fibers are characterized with respect to the influence of relative humidity changes on spectral transmission absorption and Rayleigh backscattering. The hygroscopic and thermal expansion coefficient of the fiber are determined to be CHE = (7.4 ± 0.1) $\cdot 10^{-6}$ %r.h.^−1^ and CTE = (22.7 ± 0.3) $\cdot 10^{-6}$ K^−1^, respectively. The influence of humidity on the Brillouin backscattering power and linewidth are presented for the first time to our knowledge. The Brillouin backscattering power at a pump wavelength of 1319 nm is affected by temperature and humidity. The Brillouin linewidth is observed to be a function of temperature but not of humidity. The strain coefficient of the BFS is determined to be $C_{S}=$ (−146.5 ± 0.9) MHz/% for a wavelength of 1319 nm within a strain range from 0.1% to 1.5%. The obtained results demonstrate that the humidity-induced Brillouin frequency shift is predominantly caused by the swelling of the fiber over-cladding that leads to fiber straining.

## 1. Introduction

The influence of humidity on numerous polymers has been investigated in the last decades [1,2,3,4]. The most commonly used polymer for polymer optical fibers (POFs) is polymethylmethacrylate (PMMA). PMMA absorbs up to 2.7 %wt of water depending on the level of residual monomer in the PMMA resin [5]. Among other effects, the water intake into the core of PMMA-POFs cause fiber attenuation changes [6]. Hence, PMMA-POFs were proposed for humidity sensing whether as local [6,7] or distributed sensors [8,9]. In addition, humidity-induced wavelength shifts in PMMA-POF Bragg gratings (POFBGs) were demonstrated [10,11].

Perfluorinated graded-index POFs (PFGI-POFs) use cyclic transparent optical polymer (CYTOP) as core base material and polycarbonate as over-cladding material. CYTOP fibers are characterized by lower water absorption, lower attenuation and higher bandwidth in the near infrared region in comparison to PMMA [12,13]. These beneficial characteristics are attractive for sensing applications [14,15,16,17,18] and for high-speed communication systems [19,20,21,22]. Several water absorption related effects in PFGI-POFs have been reported: (i) Considerable attenuation changes caused by water absorption into the core material of the PFGI-POF have been demonstrated by showing molecular vibration absorption in optical transmission spectra [16]. (ii) The influence of relative humidity changes on a wavelength shift has been shown in PFGI-POFBGs [18]. (iii) POFBGs inscribed in PFGI-POF without over-cladding did not show humidity-induced wavelength shifts [23]. (iv) The humidity-induced Brillouin frequency shift (BFS) in PFGI-POF was recently presented [24]. However, the reasons for the optical changes in PFGI-POFs caused by humidity remain unclear.

In a previous paper [24] we presented the linear relation between BFS and humidity changes without hysteresis effects. Furthermore, the temperature coefficient of the BFS can be considered to be independent of humidity influences [24]. In this paper we discuss the effects leading to the humidity-induced BFS and show the influence of relative humidity on the Brillouin gain spectrum (BGS). In order to characterize the fiber, spectral transmission and distributed Rayleigh measurements were performed while applying a variety of temperature and humidity levels. The properties of the BGS (backscattering power, linewidth and BFS) are presented for the first time according to our knowledge regarding their dependence on humidity. Combining the obtained measurement results with the humidity-induced BFSs of [24], a humidity-induced strain caused by a swelling of the over-cladding was identified as the dominant origin of the humidity-induced BFS.

## 2. Fiber Characterization

### 2.1. Spectral Absorption

**Figure 1:**
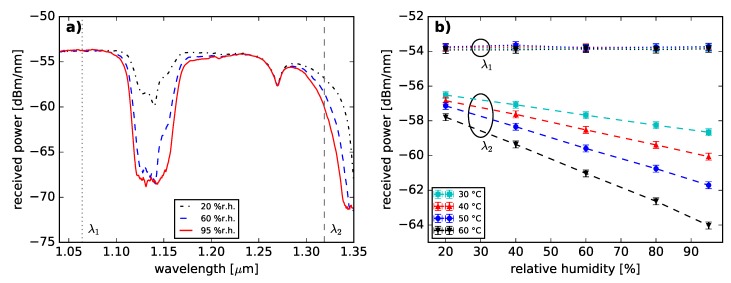
(**a**) Transmitted optical power spectra of a 200 m PFGI-POF under variation of relative humidity at 40 °C. (**b**) Transmission power as a function of humidity at specified temperatures and two wavelengths ($\lambda_{1}=1064$ nm and $\lambda_{2}=1319$ nm).

The optical transmission spectra of the PFGI-POF were measured at 4 temperature and 5 humidity values in a climate cabinet. A broadband light source (Yokogawa AQ4305) launched light into a 200 m GigaPOF-50SR by Chromis Fiberoptics (core diameter of 50 μm, 40 μm cladding layer and additional polycarbonate over-cladding resulting in a total diameter of 490 μm [25], fundamental mode diameter of 15 μm at 1319 nm [26]), which was previously annealed for 72 h at 70 °C and 95% relative humidity (r.h.) [24]. An overfilled launch condition was satisfied. The transmitted light was detected by an optical spectrum analyzer (OSA, Advantest Q8347, Tokyo, Japan). The wavelength range from 1040 nm to 1350 nm was analyzed since the fiber attenuation is significantly smaller in this specific range compared to the conventional telecommunication wavelength of 1550 nm (≈250 dB/km [13]). In addition, fiber optic components and light sources are commercially available for the wavelengths of 1064 nm and 1310 nm. Figure 1a displays selected transmission spectra at specified relative humidity values.

The absorption peaks correlate to vibrational absorption bands of the C–F bond (multiple peaks around 1130 nm [27]) and the O-H bond (center of vibration at 1383 nm [27]). The C–F bond is the characteristic bond of CYTOP [13]. Increased optical attenuations were observed for rising humidity values in both absorption bands. The optical attenuation increase with rising humidity values of the O-H peak could originate from the change of the total amount of water molecules within the fiber core material. A possible cause for the humidity-depending behavior of the carbon–fluorine bond is seen in its characteristic to be highly polarized as it attracts partially charged water molecules [28].

The wavelengths of $\lambda_{1}$ = 1064 nm and $\lambda_{2}$ = 1319 nm are marked in Figure 1a). $\lambda_{1}$ was chosen since there is little effect by vibrational absorption. Semiconductor laser diodes at $\lambda_{1}$ [29,30] might be feasible for sensing or communication applications. The humidity-induced BFS was reported at $\lambda_{2}$ [24]. The wavelength of 1319 nm will be used for all Brillouin related investigations in this paper. The corresponding transmission powers at $\lambda_{1}$ and $\lambda_{2}$ are displayed as a function of relative humidity in Figure 1b. In contrast to the transmission spectra at $\lambda_{1}$, the transmission spectra at $\lambda_{2}$ show a strong dependence on relative humidity and temperature which is consistent with [16]. Increasing relative humidity leads to increased transmission losses as shown in [16]. Furthermore, the humidity sensitivity of $\lambda_{2}$ was observed as a function of temperature. Increased temperatures result in a greater transmission loss. This behavior originates from O-H and C-F bond vibrational resonances [31].

The measurement results are consistent to [16]. In addition, the presence of water inside the fiber core material is underlined and the humidity-driven absorption of water is demonstrated. However, the exact quantity of the water uptake into the core material is not determined.

### 2.2. Rayleigh Backscattering

**Figure 2:**
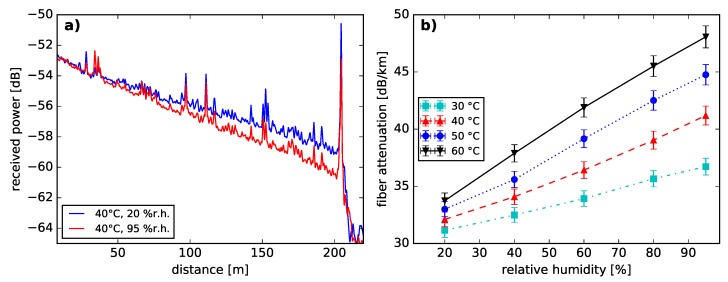
(**a**) Measured Rayleigh backscatter trace at 40 °C and 20 % r.h., 95 % r.h., respectively. (**b**) Fiber attenuation from backscatter power change as a function of relative humidity at given temperatures and a wavelength of 1306 nm. The error bars mark the threefold standard deviation from the linear regression applied on all measured data points.

Swept-wavelength interferometry (Luna OBR 4413) was used to performed the following measurements. The Luna OBR4413 has a center wavelength of 1306 nm and the sweep range of 0.8 nm was set to achieve a spatial resolution of 5 cm. In contrast to the OSA measurements, a 1 m single mode silica fiber was used to connect the PFGI-POF to the Luna device. Consequently, the measured signals are dominated by the Rayleigh scattering of the fundamental mode, due to mode selective coupling. Nevertheless, the Rayleigh influence of higher optical modes on the measurement signal can not be excluded due to the strong mode coupling of the PFGI-POF [32]. Two example backscatter traces are shown in Figure 2a.

Increasing humidity values were observed to form a sharper declining slope in the backscatter traces. A linear regression function was computed for each measured backscatter trace. The determined slope values are shown as a function of relative humidity for specified temperatures in Figure 2b. As shown in Figure 1b, the fiber attenuation increases with rising humidity and temperature values. Evidently, the fiber attenuation at 1306 nm is a function of temperature and humidity.

**Figure 3:**
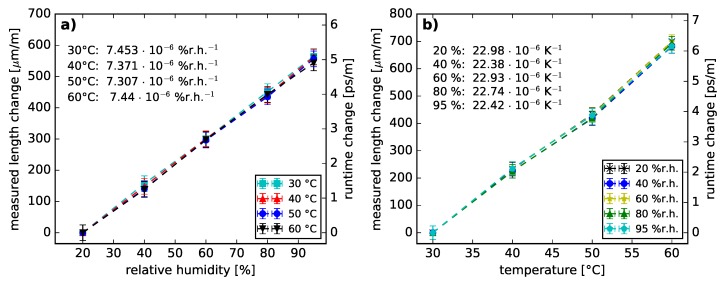
Measured relative fiber length change and optical runtime changes induced by (**a**) humidity as well as (**b**) temperature. The displayed coefficients are the slopes of the linear regression functions computed for all temperature or humidity values based on the measured fiber length changes.

In addition to the variation in fiber attenuation, a fiber length change was observed in the backscatter traces. The measured fiber length changes are plotted in relation to the obtained fiber lengths at 20%r.h. and 30 °C in Figure 3, respectively. Both data plots indicate a linear increase of measured fiber length for rising temperatures and humidities within the given measurement limits. There is no apparent cross-sensitivity of humidity and temperature change on the measured fiber length change. The computed slope values are considered as the coefficient of hygroscopic expansion (CHE) and coefficient of thermal expansion (CTE) for the tested fiber along the optical axis, respectively. The calculated expansion coefficient values are CHE = (7.4 ± 0.1) $\cdot 10^{-6}$ %r.h.^−1^ and CTE = (22.7 ± 0.3) $\cdot 10^{-6}$ K^−1^ within the given measurement ranges. The given uncertainty values are the threefold standard deviation from the linear regression applied on all measured data points, respectively. On the basis of these coefficients, the measured fiber length *l* can be described as a function of temperature and humidity as presented in Equation (1). $l_{0}$ refers to a fiber length at a given temperature and humidity. ΔT and Δh are the changes in temperature and humidity, respectively.

(1) $$\begin{array}{ccc} l(T,h) & = & l_{0}\left(1\;+\;\Delta T\cdot CTE\;+\;\Delta h\cdot CHE\right) \end{array}$$

To calculate the spatially distributed backscatter trace a constant group index of n=1.3536 [16] was used. However, the refractive index of CYTOP was reported to be a function of temperature (dn/dT = −9.7× 10^−5^ K^−1^, measured as a 3 μm thin film around 1550 nm [33]) and the absorbed water in the fiber core could cause changes in the group index. Thus, the measured fiber length should be considered as a superposition of refractive index change, temperature-induced expansion of the polymers [4] and humidity-induced swelling due to water intake [4]. Unfortunately, a distinction between the named effects is not possible using a time-of-flight-based measurement method. Therefore, Figure 3 also displays the optical runtime change as a function of relative humidity and temperature. For clarification, all measured runtime changes are reported as fiber length changes.

The humidity-induced measured fiber length change may originate from the humidity-induced change of the fiber core properties and from the humidity-depending behavior of polycarbonate [1,4], used as fiber over-cladding. As to [4], the further the humidity of the fiber surrounding increases, the more water molecules are absorbed into the fiber as a result of elevated partial vapor pressures. Since, polycarbonate tends to absorb more water than CYTOP (0.04 %wt and <0.01 %wt, respectively [12]), the dominant impact is expected for the fiber over-cladding. Consequently, a humidity-induced swelling of the polycarbonate is caused [4], resulting in an expansional straining of the fiber core along the optical axis. The described process is purely mechanical and independent on the measurement wavelength. The humidity sensitivity of the PFGI-POF strongly depends on the cladding material and its layer thickness. The measured humidity-induced fiber length change (7.4 με %r.h.^−1^) is significant compared to polyimide and acrylate coated silica single mode fibers at standard layer thickness of 15 μm (1.29 με %r.h.^−1^ and 0.2 με %r.h.^−1^, respectively [34]). Though, silica fibers with a thicker polyimide cladding demonstrate increased strain responses compared to the PFGI-POF (up to 38.5 με %r.h.^−1^ for 876 μm cladding thickness [35]).

## 3. Stimulated Brillouin Backscattering

### 3.1. Brillouin Linewidth and Backscattering Power

**Figure 4:**
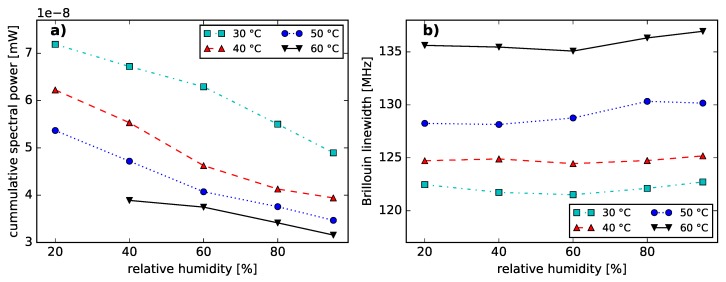
(**a**) Cumulative BGS power of the electrical signal as a function of relative humidity at specified temperatures. (**b**) Brillouin linewidth (full width at half maximum) as function of relative humidity at given temperatures.

In [24] we reported on humidity-induced BFS. On basis of the obtained noise-subtracted and non-distributed BGSs in [24], the humidity-induced changes of the stimulated Brillouin backscattering power were analyzed. A cumulative SBS power level was determined by integrating over all spectral power levels of the BGS within a 1.6 GHz span around the center frequency. Figure 4a displays the cumulative spectral power for different relative humidity settings. The SBS power decreases with increasing relative humidity and temperature.

Figure 4b shows the linewidth of the BGS from a 200 m long PFGI-POF as a function of relative humidity. This figure demonstrates no significant influence of humidity on the Brillouin linewidth. However, a sensitivity of the Brillouin linewidth on temperature was observed, which is consistent to [36]. The Brillouin linewidth in PFGI-POFs increases with increasing temperatures, whereas the Brillouin linewidth in silica fibers decreases with increasing temperatures in the same temperature range [37].

This phenomenon can be explained when taking into account the thermoplastic properties of both, CYTOP and polycarbonate [13,38]. The polymer chains are associated through intermolecular forces, which weaken with increasing temperatures [38,39]. Thus, an increased moldability and a reduced resistance for external deformation is achieved by heating [38] and can be considered as a reduced elastic modulus with rising temperatures. The temperature dependency of the elastic modulus in PFGI-POF was demonstrated in [40]. The cross effect of temperature and strain on the BFS presented in [41] can be considered as temperature-induced changes of the elastic modulus. Hence, an elevated temperature can be considered to lead to a reduced phonon lifetime and consequently to a greater Brillouin linewidth [42]. This spectral spreading of the BGS with risen temperatures causes a lowered Brillouin gain coefficient [42]. Consequently, the **temperature-induced reduction of the SBS power level** can be considered to be dominantly caused by the thermoplastic characteristic of CYTOP and the vibrational absorption dependence (see Figure 1b and Figure 2b).

The origin of the **humidity-induced SBS power reduction** can be largely attributed to the vibrational absorption of the fiber core material. While increased humidity levels lead to higher fiber losses (see Figure 1b and Figure 2b), the effective length of the PFGI-POF decreases [42]. Consequently, the Brillouin pump threshold rises [42]. Therefore, we expect the SBS interaction to occur on a shorter fiber length. In addition, the Brillouin backscattered light is attenuated. However, humidity-induced changes of the Brillouin gain coefficient should be further investigated in the future.

### 3.2. Brillouin Frequency Shift

Commonly the BFS is described by Equation (2) [42,43]. $f_{B}$ is the value of the Brillouin frequency shift, λ the wavelength of the laser, *n* and $v_{a}$ are the refractive index and the speed of sound of the guiding medium. As the light-induced acoustic waves in optical fibers are described as longitudinal waves [42,44], the velocity of the acoustic wave is expressed as $v_{a}=\sqrt{\epsilon /\rho }$. ϵ is the elastic modulus and ρ the density of the guiding medium [42].

(2) $$f_{B}=\frac{2n\;v_{a}}{\lambda }=\frac{2n\sqrt{\epsilon /\rho }}{\lambda }$$

According to (2) the humidity-induced BFS can be explained by changes in the core material parameters *n*, ϵ and ρ. The humidity variations affect these parameters directly due to water absorption into the fiber core or indirectly as a result of humidity-induced fiber strain as described in the previous section. To be able to discuss the humidity-induced fiber strain, the strain coefficient of the BFS was determined, since it is unknown for a wavelength of λ=1319 nm.

**Figure 5:**
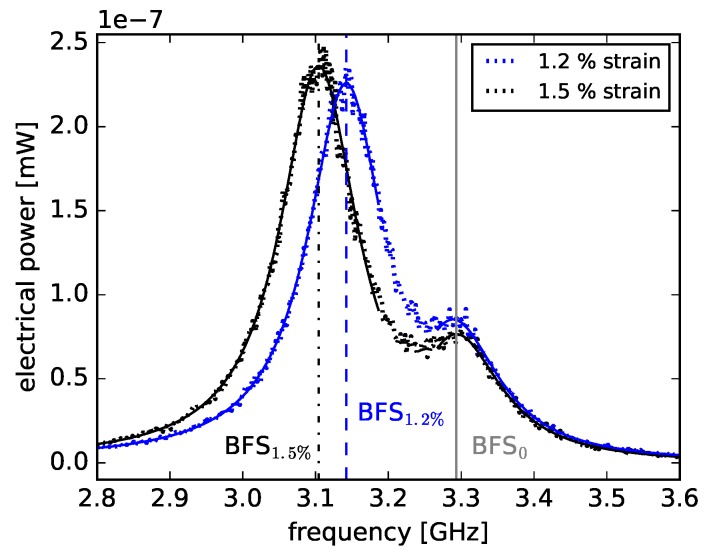
BGSs of a 200 m PFGI-POF with applied strain values of 1.2% and 1.5% in the first 90 m, measured by an electrical spectrum analyzer. The regression functions of the strained and non-strain fiber segments are shown as well as their corresponding BFS.

Using the experimental setup of [24] the BFS was measured while applying uniform strain to the first 90 m of a 200 m PFGI-POF. The fiber was strained using two fiber spools, whereby one was static and the second one was mounted on a stepper motor, controlling the distance between both spools. The applied strain was considered uniform since the spools were mounted to allow a rotation around their middle axis. The spools were simultaneously rotated half a turn before each measurement to release tension inhomogeneities. The first 90 m of the fiber were wrapped between the spools whereas the remaining fiber was freely placed to prevent further strain-induced BFSs. This fiber segmentation allows a temperature-compensated determination of the BFS for all strain values greater than 0.6%. Figure 5 shows two measured BGSs with applied strain values of 1.2% and 1.5%.

The BGS relating to the first 90 m fiber segment is affected by temperature fluctuations and strain changes, whereas the BGS of the non-strained segment is dominantly influenced by temperature fluctuations. The BGSs of the two fiber segments are separating from each other, since the applied strain causes a greater BFS change compared to the BFS change caused by temperature fluctuations. Thus, two Lorentzian regression functions were computed for each measured electrical spectrum to determine the BFS corresponding to each fiber segment (see BFS_0_, BFS_1.2%_ and BFS_1.5%_ in Figure 5). The strain-induced BFS change is determined by subtracting the BFS of the strained section from the BFS of the unstrained section (for example BFS_1.2%_− BFS_0_).

However, the spectral separation of the two BGSs was not feasible for strain values below 0.6%, as the two BGSs are superposed. Therefore, a Lorentzian regression calculation was applied to the complete measured spectrum. The determination of strain-related BFS using the complete spectrum was possible since the SBS power of the strained section was dominant compared to the unstrained section. Unfortunately, the obtained BFS contains strain and temperature information which increases the uncertainty of the measurements compared to the BGS separation approach. The strain-induced BFS change was determined by subtracting the BFS of the strained fiber from the BFS of the unstrained fiber. All strain measurements were performed at room temperature. The maximum temperature drift during a single measurement was observed to be ±0.5 K.

**Figure 6:**
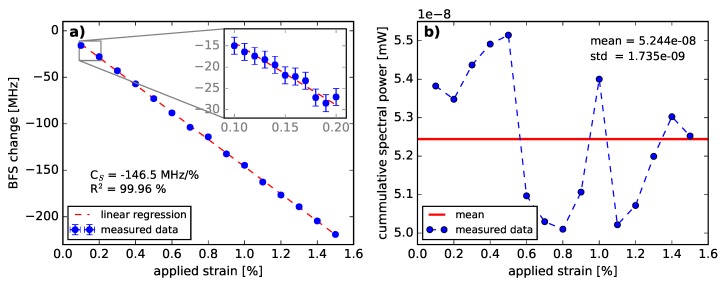
(**a**) Strain-induced change of the Brillouin frequency shift as a function of strain and the calculated strain coefficient of the BFS in the range of 0.1% to 1.5 %. The inset provides a closer view on the measurement data in the range of 0.1% to 0.2 %. (**b**) Cumulative BGS power of the whole measured spectrum as a function of applied strain.

The BFS change caused by applied strain is plotted in Figure 6a. A linear decrease of the BFS with applied strain was observed, which is consistent to [45]. The strain coefficient of the BFS was calculated to be $C_{S}=$ (−146.5 ± 0.9) MHz/% in the strain range from 0.1% to 1.5%. Taking into account the $\lambda^{-1}$ relationship of the Brillouin coefficients [46], the determined strain coefficient value at 1319 nm are in good agreement with the previously reported value of $C_{S}=$ −121.8 MHz/% at 1550 nm [45] in the same strain range. The maximum fiber strain applied in the experiment was chosen to be 1.5 %, since strains greater than 2 % result in a non-linear BFS behavior [47]. Figure 6b shows the cumulative spectral power of the noise-subtracted BGSs within a frequency span of 1.6 GHz plotted against applied strain. A non-linear course over strain was observed. The power fluctuations are associated with laser and SBS power fluctuations. The latter are the consequence of polarization effects. Thus, strain up to 1.5% does not significantly affect the cumulative BGS power.

**Figure 7:**
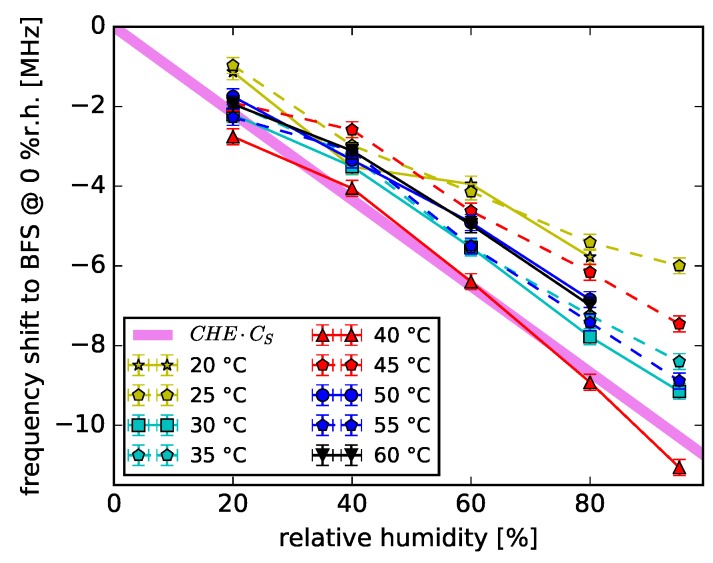
BFS as a function of relative humidity at specified temperatures. The BFS is given as a difference to the theoretical BFS at each corresponding temperature and 0 %r.h.

In a previous publication [24] we demonstrated, that humidity and temperature are two mutually independent influences on the BFS. Thus, the temperature BFSs can be separated from the measured BFS to study the humidity-induced changes more closely. The BFS results of our humidity investigation [24] are plotted as a function of relative humidity for different temperatures in Figure 7.

At all temperatures a BFS decrease with rising humidity values was observed. As previously shown in Section 2.2, humidity causes a measured fiber length change. Consequently, the coefficients CHE = 7.4 $\cdot 10^{-6}$ %r.h.^−1^ and $C_{S}$ = −146.5 MHz/% were used to calculate a theoretical BFS coefficient describing the fiber strain provoked by humidity ($CHE\cdot C_{S}$ = (−108.4 ± 9.0) kHz/%r.h). Under the assumption that the measured length change due to humidity change can be predominantly contributed to the swelling of the fiber over-cladding, we plotted the swelling strain-equivalent dependence $CHE\cdot C_{S}$ in Figure 7. This assumption is backed up by the comparison of [18] and [23]. In [18] the POFBG inscribed into a PFGI-POF demonstrated a wavelength shift increase with rising humidity (14.7 pm/%r.h.). Whereas the POFBG in [23] was inscribed into a PFGI-POF without over-cladding demonstrating no wavelength shifts for 40%r.h. change. As the $CHE\cdot C_{S}$ and the measured BFSs are in good agreement, the humidity-induced strain can be considered as the dominant cause of the humidity-induced BFS. In addition, the theoretical coefficient $CHE\cdot C_{S}$ and therefore the BFS change caused by cladding-induced strain is independent on temperature influences within the measured temperature and humidity ranges.

The isothermal BFS graphs demonstrate divergence from each other with rising humidity values. However, a detailed analysis of the temperature behavior of the humidity-induced core property changes apart of fiber strain can not be given based on this data set. The coefficients $C_{S}$ and CHE were determined at different wavelengths (1319 nm and 1306 nm, respectively). These wavelengths are close to each other, though the exact influence of the wavelength difference on the humidity-induced strain coefficient and its uncertainty should be further investigated. Thus, a temperature dependence of the humidity-induced changes of the core material parameters can not be excluded as it is indicated in the analytical description for the humidity-induced BFS [24].

When taking into account the fiber strain and the change of the refractive index and/or density, the lack of a humidity-induced BFS hysteresis presented in [24] can be explained. The humidity-induced strain is within the fully elastic strain limit of the PFGI-POF (up to 2.5% [16]). Furthermore, the water absorption into the fiber can be considered fully reversible without any damages for an annealed PFGI-POF as described for PMMA-POFs in [48].

## 4. Conclusions

In this paper the influence of humidity on SBS in PFGI-POF is described. Fiber characterization measurements demonstrated the fiber attenuation to be a function of wavelength, temperature and humidity due to vibrational absorptions. Furthermore, the fiber expansion coefficients for humidity and temperature are determined to be CHE = (7.4 ± 0.1) $\times 10^{-6}$ % r.h.^−1^ and $CTE\;=\;\left(22.7\;\pm \;0.3\right)\;\times \;10^{-6}\;K^{-1}$. The cumulative Brillouin backscattering power at a pump wavelength of 1319 nm is affected by vibrational absorption and therefore shows dependence on temperature and humidity. The Brillouin linewidth is observed to be a function of temperature, but not of humidity. Thus, the elastic modulus of the PFGI-POF can be considered to be affected by temperature and not by humidity. The strain coefficient of the BFS is determined to be $C_{S}=$ (−146.5 ± 0.9) MHz/% for a wavelength of 1319 nm within a strain range from 0.1% to 1.5%. The obtained results indicate that the humidity-induced BFS is predominantly caused by the humidity-caused swelling of the fiber over-cladding that leads to fiber straining. PFGI-POFs demonstrate a greater humidity-induced strain response compared to standard silica fibers coated with polyimide and acrylate. All presented results are also relevant for other temperature and humidity fiber sensors in PFGI-POF such as fiber Bragg gratings and distributed sensors based on Rayleigh or stimulated Brillouin scattering.

## Abbreviations

The following abbreviations are used in this manuscript:

POF	polymer optical fiber
PMMA	polymethylmethacrylate
PMMA-POF	POF based on PMMA
POFBG	polymer optical fiber Bragg grating
PFGI-POF	perfluorinated graded-index POF
CYTOP	cyclic transparent optical polymer
BFS	Brillouin frequency shift
SBS	stimulated Brillouin scattering
OSA	optical spectrum analyzer
BGS	Brillouin gain spectrum
CHE	coefficient of hygroscopic expansion
CTE	coefficient of thermal expansion
